# Radiotherapy improves survival in early stage extranodal natural killer/T cell lymphoma patients receiving asparaginase-based chemotherapy

**DOI:** 10.18632/oncotarget.14006

**Published:** 2016-12-17

**Authors:** Yi-Yang Li, Ling-Ling Feng, Shao-Qing Niu, Han-Yu Wang, Lu-Lu Zhang, Liang Wang, Zhong-Jun Xia, Hui-Qiang Huang, Yun-Fei Xia, Yu-Jing Zhang, Xi-Cheng Wang

**Affiliations:** ^1^ Department of Oncology, the First affiliated Hospital of Guangdong Pharmaceutical University, Guangdong 510080, People's Republic of China; ^2^ State Key Laboratory of Oncology in South China, Collaborative Innovation Center for Cancer Medicine, Guangzhou, Guangdong 510060, People's Republic of China; ^3^ Department of Radiation Oncology, Sun Yat-sen University Cancer Center, Guangzhou, Guangdong 510060, People's Republic of China; ^4^ Department of Radiation Oncology, First Affiliated Hospital of Sun Yat-sen University, Guangzhou, Guangdong 510060, People's Republic of China; ^5^ Department of Hematologic Oncology, Sun Yat-sen University Cancer Center, Guangzhou, Guangdong 5100600, People's Republic of China; ^6^ Department of Medical Oncology, Sun Yat-sen University Cancer Center, Guangzhou, Guangdong 510060, People's Republic of China

**Keywords:** extranodal natural killer/T cell lymphoma, non-Hodgkin lymphoma, asparaginase, radiotherapy, chemotherapy

## Abstract

This study retrospectively investigated asparaginase-based chemotherapy treatment outcomes with or without radiotherapy in 143 patients with stage I_E_–II_E_ extranodal natural killer/T cell lymphoma (ENKTCL). All patients received a median of three cycles of asparaginase-based chemotherapy, while 121 patients received radiotherapy following the chemotherapy. The complete remission (CR) rate for all patients post-chemotherapy was 58.7%, and rose to 73.4% by the end of treatment. Patients who received radiotherapy achieved better survival outcomes than those who did not (89.7% vs. 49.0% for 2-year overall survival (OS), *P*<0.001; 86.8% vs. 37.4% for 2-year progression-free survival (PFS), *P*<0.001). Additionally, even patients who achieved CR post-chemotherapy exhibited differential survival rates with or without radiotherapy (90.8% vs. 60% for 2-year OS, *P*=0.006; 86.1% vs. 60% for 2-year PFS, *P*=0.044). Multivariate analysis revealed that radiotherapy was an independent factor favoring OS (HR=0.098, 95%CI=0.031–0.314, *P*=0.001) and PFS (HR=0.156, 95%CI=0.062–0.396, *P*=0.001). Thus, radiotherapy is recommended for stage I_E–_II_E_ ENKTCL patients treated with asparaginase-based chemotherapy, even in cases of CR following chemotherapy.

## INTRODUCTION

Extranodal natural killer/T cell lymphoma (ENKTCL), a type of non-Hodgkin lymphoma (NHL), is rare in western countries, but prevalent in South America and East Asia [1]. In China, ENKTCL occurs mainly in the upper aerodigestive tract and accounts for 2 to 10% of primary NHL cases in the nasal cavity [2]. Compared with other forms of NHL, it is characterized by extensive local invasion, association with Epstein-Barr virus (EBV) infection, necrosis with angiodestruction and offensive odor, chemoresistance, and radiosensitivity [3–8].

Unlike other NHL subtypes, conventional doxorubicin-based chemotherapies, including CHOP (cyclophosphamide, doxorubicin, vincristine, and prednisone) and EPOCH (etoposide, vincristine, doxorubicin, cyclophosphamide and prednisone), do not provide good ENKTCL treatment outcomes, due to multidrug-resistant (MDR) gene and P-glycoprotein overexpression in tumor cells [9–14]. As a result of low anthracyclin-based chemotherapy efficacy, radiotherapy plays a vital role in treating early stage ENKTCL. L-asparaginase recently showed promising results in overcoming ENKTCL chemoresistance [15–19]. Asparaginase-based regimens, like combined dexamethasone, methotrexate, ifosfamide, L-asparaginase and etoposide (SMILE), and combined asparaginase, methotrexate and dexamethasone (AspaMetDex), were effective against relapsed and refractory ENKTCL [16, 17]. The GELOX regimen, which consists of gemcitabine, oxaliplatin, and L-asparaginase, is a new induction chemotherapy. GELOX provided superior treatment outcomes and safety compared with doxorubicin-based chemotherapy in patients with previously-untreated stage I_E_–II_E_ ENKTCL, with both 2-year overall survival (OS) and progression-free survival (PFS) reaching 86% [18, 19]. However, the value of radiotherapy with asparaginase-based chemotherapy remains unexplored. We conducted this retrospective study to assess outcomes in I_E_-II_E_ stage ENKTCL patients treated with asparaginase-based induction chemotherapy with or without radiotherapy.

## RESULTS

### Patient characteristics

Patient characteristics are presented in Table 1. Patient age ranged from 13 to 79 years with a median age of 45, and 25 were aged >60. 108/143 patients presented with disease located in the nasal cavity, while disease was located in Waldeyer's ring in 37/143 cases. Our study employed the Stage-modified International Prognostic Index (mIPI), in which adverse prognostic factors include age >60, Ann Arbor stage II, elevated LDH level, >1 extranodal sites involved, and Eastern Cooperative Oncology Group (ECOG) performance score 2–4. Numbers of patients with mIPI 0~1, 2, 3 and 4~5 were 77, 47, 15 and 4, respectively.

**Table 1:** Clinical characteristics of patients according to radiotherapy

Characteristic	All patients			The CR patients after CT		
	RT group(n=121)	No-RT group(n=22)	P	RT group(n=71)	No-RT group(n=13)	P
age			0.001			0.001
>60	15(12.3%)	10(45.5%)		8(11.3%)	7(53.8%)	
≤60	106(87.7%)	12(54.5%)		63(88.7%)	6(46.2%)	
gender			0.883			0.472
male	75(62%)	14(63.6%)		44(62%)	10(76.9%)	
female	46(38%)	8(36.4%)		27(38%)	3(23.1%)	
Primary site			0.836			0.453
nasal cavity	91(75.2%)	17(77.3%)		56(78.9%)	12(92.3%)	
Waldeyer's ring	30(24.8%)	5(22.7%)		15(21.1%)	1(7.7%)	
B symptom			0.694			1.000
present	55(45.5%)	11(50%)		35(47.3%)	6(46.2%)	
absent	66(54.5%)	11(50%)		36(50.7%)	7(53.8%)	
Local invasion			0.941			0.472
yes	78(64.5%)	14(63.6%)		36(50.7%)	8(61.5%)	
no	43(35.5%)	8(36.4%)		35(47.3%)	5(38.5%)	
Ann Arbor stage			0.250			0.650
I	71(58.7%)	10(45.5%)		43(60.6%)	7(53.8%)	
II	50(41.3%)	12(54.5%)		28(39.4%)	6(46.2%)	
LDH level			0.827			0.751
normal	96(79.3%)	17(77.3%)		54(76.1%)	11(84.6%)	
elevated	25(20.7%)	5(22.7%)		17(23.9%)	2(15.4%)	
ECOG score			0.045			0.329
0~1	106(87.6%)	15(68.2%)		61(85.9%)	13(100%)	
2	15(12.4%)	7(31.8%)		10(14.1%)	0(0%)	
mIPI			0.114			0.060
0~1	67(55.4%)	10(45.5%)		41(57.7%)	5(38.5%)	
2	42(34.7%)	5(22.7%)		22(33.8%)	3(23.1%)	
3	10(8.3%)	5(22.7%)		5(7.1%)	2(15.3%)	
4~5	2(1.6%)	2(9.1%)		3(1.4%)	3(23.1%)	

Abbreviations: LDH, lactate dehydrogenase; ECOG, Eastern Cooperative Oncology Group; mIPI, stage-modified International Prognostic Index.

We compared patient baseline characteristics and found that the radiotherapy-treated group included more individuals aged <60 and with lower ECOG scores than the group that did not receive radiotherapy (Table 1).

### Response to treatment

58.7% (84/143) of patients achieved CR after asparaginase-based induction chemotherapy, and the remaining patients experienced PR (40/143, 28.0%), SD (12/143, 8.4%), or PD (7/143, 4.9%). Among the seven progressed patients, three presented with enlargement of local neoplasm, and four with distant metastasis, including to skin, lungs and bone. No patients died during treatment. At the end of chemotherapy, 53 patients developed grade 3 or 4 toxicities, mainly leukopenia, thrombocytopenia and increased transaminases. Three patients delayed chemotherapy as a result of severe leukopenia or impaired liver function.

Due to systemic progression, chemotherapy-induced toxicities, or fear of irradiation, 22 patients rejected sequenced radiotherapy, including 13 CRs after chemotherapy. Radiotherapy was delivered to 121 patients, including 71 CRs, 39 PRs, nine SDs and two PDs after asparaginase-based chemotherapy. 36 patients received consolidation chemotherapy following radiotherapy. Radiotherapy improved treatment response in 29 patients (21 from PR to CR, six from SD to CR/PR, two from PD to PR/SD). At the end of treatment, the CR rate reached 73.4% (105/143).

### Survival and prognostic analysis

**Figure 1:**
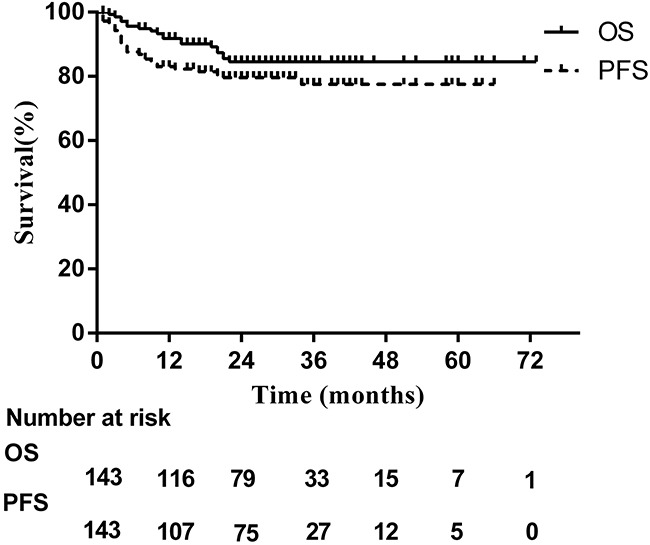
Survival curves for all patients treated with asparaginase-based chemotherapy

**Figure 2:**
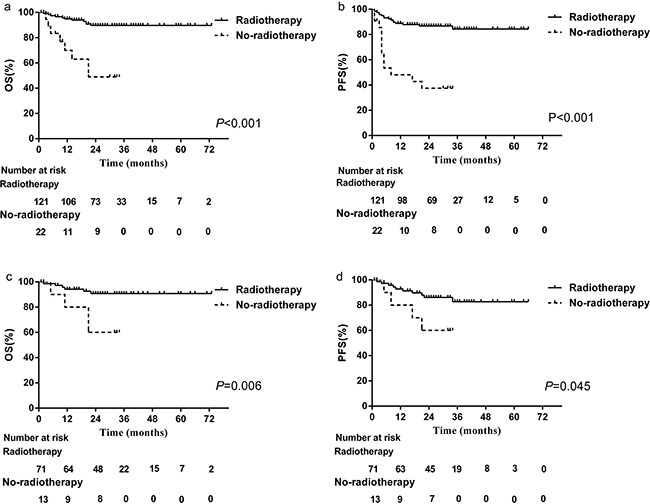
Patient OS and PFS All patients treated with **A**. or without **B**. radiotherapy. CR patients treated with **C**. or without **D**. radiotherapy.

By the end of March 2016, 91% of the patients were still included in the follow-up, with a mean follow-up time among the surviving patients of 28 months. The 2-year OS and PFS for the cohort were84.6% and 79.6%,respectively (Figure 1). 27 patients had died, all of disease progression. Compared to those who did not receive radiotherapy, patients treated with radiotherapy achieved superior survival in terms of OS (89.7% vs. 49.0%, *P*<0.001, Figure 2A) and PFS (86.8% vs. 37.4%, *P<*0.001, Figure 2B). However, baseline characteristics may have factored in these results. Radiotherapy-treated patients tended to exhibit more favorable factors, such as younger age and lower ECOG score, and responded better to asparaginase-based chemotherapy. To more accurately determine the effects of radiotherapy on survival, subgroup analysis was conducted according to chemotherapy response. Even for those who achieved CR after chemotherapy, radiotherapy provided a survival benefit: 2-year OS was 90.8% for patients treated with radiotherapy, compared with 60.0% for those who were not (*P*=0.006, Figure 2C). Similarly, PFS was 86.1% for radiotherapy compared with 60.0% for no radiotherapy (*P*=0.044, Figure 2D).

Univariate analysis of patient clinical characteristics revealed that stage II disease and no radiotherapy were significant factors adversely affecting both OS and PFS. The multivariate Cox regression model showed that radiotherapy was an independent favorable prognostic factor for OS (HR=0.174, 95%CI=0.069–0.437, *P*<0.001) and PFS (HR=0.179, 95%CI=0.085–0.376, *P*<0.001) (Table 2). Ann Arbor stage was not an independent OS (*P*=0.090) or PFS (*P*=0.075) prognostic factor. Among CR patients, univariate analysis suggested that radiotherapy was the only factor associated with improved OS (P=0.007) and PFS (P=0.045); thus no multivariate analysis was performed.

**Table 2:** Univariate and multivariate analysis of prognostic factors and patient characteristics

Characteristic	Univariate analysis		Multivariate analysis			
	OS	PFS	OS		PFS	
	*P* value	*P* value	HR(95%CI)	*P* value	HR(95%CI)	*P* value
Age<60	0.224	0.320				
Male gender	0.656	0.701				
B symptom	0.986	0.707				
Nasal cavity origin	0.672	0.551				
Local invasion	0.089	0.069				
ECOG score >2	0.175	0.264				
Ann Arbor stage II	0.045	0.031	2.254(0.880-5.773)	0.090	1.954(0.934-4.085)	0.075
Elevated LDH level	0.575	0.787				
RT	0.001	0.001	0.174(0.069-0.437)	0.001	0.179(0.085-0.376)	0.001

Abbreviations: OS, overall survival; PFS, progression-free survival; LDH, lactate dehydrogenase; CT, chemotherapy; RT, radiotherapy.

## DISCUSSION

Due to the rarity and heterogeneity of ENKTCL, and lack of prospective trial data, no clinical treatment consensus has yet been reached. Based on diffuse large B-cell lymphoma (DLBCL) results, early-stage ENKTCL was previously treated with doxorubicin-based chemotherapy with or without radiotherapy. However, ENKTCL demonstrates resistance to doxorubicin-based chemotherapy. In previous studies, the 2-year OS of patients treated with CHOP ranged from 44.2 to 78% [9–12], and changing the drug delivery schedule did not greatly improve treatment outcomes. Huang, *et al*. reported a CR rate of 75% and a 3-year OS rate of 75% after EPOCH induction chemotherapy followed by radiotherapy [13]. ENKTCL is highly resistant to these conventional doxorubicin-based chemotherapy regimens, and the identification of a more effective chemotherapy regimen is imperative.

L-asparaginase, which was first proposed for ENKTCL treatment by Yong, *et al*., can overcome ENKTCL chemotherapy resistance and has improved patient outcomes [15–19, 20–22]. In 39 refractory and relapsed ENKTCL patients receiving radiation after vincristine, prednisolone and asparaginase-based chemotherapy, the objective response rate after treatment and 5-year OS reached 82.2% and 66.9%, respectively [15]. Jiang, *et al*. conducted a small cohort phase II study, in which vincristine, prednisolone and asparaginase-based chemotherapy plus radiotherapy was administered to 26 early stage previously-untreated ENKTCL patients, yielding a CR rate of 80.8% and 2-year OS and PFS of 88.5% and 80.6%, respectively [21]. Another prospective study from our center treated 27 early stage patients with GELOX induction chemotherapy followed by definitive radiotherapy. The results were promising, with 74.1% CR and 86% 2-year OS [18].

As a retrospective study, the present work analyzed the largest number of early stage ENKTCL patients treated with asparaginase-based chemotherapy to date. In our study, 73.4% CR was achieved after treatment, and 2-year OS and PFS were 84.6% and 79.6%, respectively. Compared with CHOP or EPOCH regimens, GELOX improved 3-year OS (54.0%, 54.0%, and 87.0%, respectively for OS, *P*<0.05) and PFS (43.0%, 50.0%, and 72.0%, respectively for PFS, *P*<0.05). These results demonstrate that asparaginase-based chemotherapy yields highly promising treatment outcomes, and is an effective treatment option for stage I_E_–II_E_ ENKTCL.

Due to the high efficacy of asparaginase-based chemotherapy, radiotherapy had not yet been assessed in combination with this treatment in ENKTCL patients. In our study, we first compared survival in radiotherapy-treated patients to those who received chemotherapy alone, and found superior OS and PFS in patients treated with radiotherapy (*P*<0.001). Notably, post-chemotheraputic radiotherapy improved patient response to initial chemotherapy, and caused 49.2% of patients (29/59) to transition from PR to CR, or SD/PD to PR/CR. This suggested that appropriate irradiation may partially or completely eradicate residual disease after initial asparaginase-based chemotherapy, and could increase survival as compared to chemotherapy alone. However, this discrepancy was biased by confounding factors, such as differential response after chemotherapy between the two groups. To further assess the effect of radiotherapy following chemotherapy, we performed a subgroup analysis of the 84 patients who exhibited CR after asparaginase-based chemotherapy. Patients who received radiotherapy experienced a survival benefit compared to those who did not (2-year OS 90.8% vs. 60.0%, *P*=0.006; 2-year PFS 86.1% vs. 60.0%, *P*=0.044). Multivariate analyses further confirmed radiotherapy as an independent prognostic factor for PFS. This indicated that benefits from asparaginase-based regimens were limited, and radiotherapy was still necessary in early stage ENKTCL. Appropriately timing for radiotherapy delivery is critical to improved treatment outcome. Zang, *et al*. divided 64 stage I-II ENKTCL patients treated with asparaginase-based regimens (CHOP-L or SMILE) followed by radiotherapy into two groups according to cycles of chemotherapy: the early radiotherapy group (no more than three cycles of initial asparaginase-based regimens before radiotherapy) and the late radiotherapy group (more than six cycles of initial chemotherapy followed by radiotherapy) [8]. Three-year OS and PFS were 84.2 and 74.3%, respectively, for early radiotherapy and 57.6 and 55.9%, respectively, for late radiotherapy. Differences between early and later radiotherapy survival outcomes were significant (*P*=0.027 for OS, *P*=0.034 for PFS). According to these results, early radiotherapy should be used in ENKTCL patients in combination with asparaginase-based regimens. Our data suggest that short-term asparaginase-based chemotherapy followed by radiotherapy is recommended for early stage ENKTCL treatment.

Some limitations were present in our study. PET-CT is a valuable staging and treatment evaluation tool for ENKTCL. Lacking PET-CT, PR patients may be designated mistakenly as CR according to MRI results. In such cases, survival benefit from radiotherapy could be exaggerated, impairing the credibility of CR patient results to some extent. Additionally, given the retrospective nature of our study, certain patient characteristics differed between the radiotherapy-treated versus no radiotherapy groups. For example, a higher proportion of patients >60 years of age who experienced CR after chemotherapy refused further radiotherapy. However, a multivariate analysis found that age was not an independent prognostic factor in our study, and thus had little impact on our radiotherapy-related survival benefit analyses. Still, analyses of larger case numbers, and prospective, randomized studies should be performed to confirm our results.

In conclusion, our results showed that radiotherapy was an independent favorable prognostic factor for OS and PFS in ENKTCL patients. Thus, radiotherapy is recommended for stage I_E–_II_E_ ENKTCL patients treated with asparaginase-based chemotherapy, even in cases with CR to asparaginase-based chemotherapy.

## MATERIALS AND METHODS

### Patients

A total of 165 ENKTL patients treated at Sun Yat-sen University Cancer Center between June 2007 and March 2015 were retrospectively enrolled using the following criteria: (1) diagnosis of ENKTCL with pathologically confirmed evidence according to the WHO classification of lymphomas [1]; (2) primary sites in upper aerodigestive tract; (3) newly diagnosed patients with Ann Arbor stage I_E_ to II_E_ disease; (4) asparaginase-based chemotherapy with or without radiotherapy; (5) patients with complete follow-up data. Informed consent for the collection of medical information was received from patients at the first visit, and the ethics committee of Sun Yat-sen University Cancer Center approved this study. Additionally, all procedures performed involving human participants were in accordance with the 1964 Helsinki declaration and its later amendments, or comparable ethical standards.

Disease stage was evaluated based on a thorough review of patient medical history, physical examination, laboratory data (such as lactate dehydrogenase (LDH) levels), direct or indirect nasopharyngoscopy, head and neck magnetic resonance imaging, computed tomography scan of the chest, abdomen, and pelvis, and bone marrow aspiration or biopsy. As a staging measure, positron emission tomography-computed tomography (PET-CT) was performed in 67 patients. All patients were staged in accordance with the Ann Arbor system. Local invasion was defined when tumors spanned neighboring structures by contiguous spread, such as nasal skin, paranasal sinus, orbit, and hard or soft palate. B symptoms were defined as unexplained recurrent fever (temperature above 38°C), night sweats, and unexplained weight loss of more than 10% in the 6 months before diagnosis.

### Treatment

All patients were first treated with chemotherapy at our institution. Regimens varied according to the attending physicians, and included GELOX (gemcitabine, oxaliplatin, pegaspargase), AspMetDex (L-asparaginase, methotrexate, dexamethasone), SMILE (dexamethasone, methotrexate, ifosfamide, L-asparaginase, etoposide), CHOP-L (cyclophosphamide, doxorubicin, vincristine, prednisone, L-asparaginase), LVP (L-asparaginase, vincristine, prednisone), and DDGP (gemcitabine, pegaspargase, cisplatin, dexamethasone) (Table 3). All 143 patients received 1–6 (median, 3) cycles of asparaginase-based chemotherapy. Patients who achieved complete response (CR), partial response (PR), and stable disease (SD) were referred for radiotherapy, which was also suggested as palliative treatment for PR patients with local progressed disease. 121 patients received radiotherapy after induction chemotherapy, while radiotherapy was terminated in the remaining 22 patients as a result of disease progression, chemotherapy-induced toxicities or personal reasons. Consolidation asparaginase-based chemotherapy was delivered to 37/121 patients after radiotherapy.

**Table 3:** Chemotherapy regimens of all 165 patients according to chemotherapy

Regimens and drugs	Dose (mg/m^2^)	Days	route	Cases
P-GEMOX		21		104
Gemcitabine	800 mg/m^2^	1,8	IV	
Oxaliplatin	100 mg/m^2^	1	IV	
Pegaspargase	2500 U/m2	1	IM	
AspMetDex		21		4
L-asparaginase	6,000 U/m^2^	2,4,6,8	IV	
methotrexate	3000 mg/m^2^	1	IV	
dexamethasone	40mg	1-4	PO	
SMILE		28		3
Dexamethasone	40 mg	2-4	PO	
Methotrexate	2 g/m^2^	1	IV	
Ifosfamide	1,500 mg/m^2^	2-4	IV	
L-asparaginase	6,000 U/m^2^	8, 10, 12, 14, 16, 18, 20	IV	
Etoposide	100 mg/m^2^	2-4	IV	
CHOP-L		21		22
Cyclophosphamide	750	1	IV	
Doxorubicin	50	1	IV	
Vincristine	1.4	1	IV	
Prednisone	60	1-5	PO	
L-asparaginase	6,000 U/m^2^	1-7	IV	
LVP		21		12
L-asparaginase	6,000 U/m^2^	1-5	IV	
Vincristine	1.4	1	IV	
Prednisone	100	1-5	PO	
DDGP		21		20
Gemcitabine	800 mg/m^2^	1,8	IV	
Pegaspargase	2500 U/m2	1	IM	
cisplatin	20	1-4	IV	
dexamethasone	15mg/m^2^	1-5	IV	

Abbreviations: ENKTL, extranodal natural killer/T cell lymphoma; ASP, asparaginase.

Following chemotherapy, extended involved-field radiotherapy was delivered to 121 patients, 22 via three-dimensional conformal radiotherapy (3DCRT) and 99 via intensity-modulated radiotherapy (IMRT) [23]. Administered with a 6-MV or 8-MV linear accelerator, the radiotherapy dose was 46–66 Gy/20–30 fractions (median dose, 54.6 Gy/26 fractions).

### Response and toxicity assessment

In reference of revised response criteria for NHL [24], treatment response was evaluated before and after radiotherapy according to the results of physical examination, EBV DNA copy number, indirect nasopharyngoscopy, and imaging examination (87 patients) or PET-CT (56 patients). CR was defined as disappearance of all detectable disease, including PET-CT negative or regression to normal size on MRI. All adverse effects of chemotherapy were graded in accordance with version 4.0 of the National Cancer Institute Common Terminology Criteria for Adverse Events.

### Statistical analysis

Follow-up time was measured from treatment initiation to the patient's death or last follow-up visit. PFS was calculated from the date of first treatment to the date of first documented disease progression or relapse, or the date of last follow-up visit. OS was defined as the interval from the time of first treatment to the time of death from any cause or the time of last follow-up visit.

Statistical analyses were performed using SPSS 19.0 software. Comparisons of qualitative data were performed by *χ*^2^ analysis. The Kaplan–Meier method was applied to estimate survival, and differences in survival curves were assessed using the log-rank test. Multivariate analysis was performed using the stepwise forward Cox model. A two-sided *P*<0.5 was considered statistically significant.
